# Abnormal uterine bleeding control with combined oral contraceptives: a review comparing ethinylestradiol and natural hormones

**DOI:** 10.61622/rbgo/2025rbgo84

**Published:** 2025-10-21

**Authors:** Júlia de Oliveira Nadaleto, Juliana Tamada Ebenur, Diandra Ravidá Alves de Macedo, Maria Fernanda Sanfins Marrelli, Lívia Bonin Ferreira, Gabriela Pravatta-Rezende

**Affiliations:** 1 Faculdade de Medicina de Jundiaí Jundiaí SP Brazil Faculdade de Medicina de Jundiaí, Jundiaí, SP, Brazil; 2 Universidade Estadual de Campinas Faculdade de Ciências Médicas Departamento de Ginecologia e Obstetrícia Campinas SP Brazil Departamento de Ginecologia e Obstetrícia, Faculdade de Ciências Médicas, Universidade Estadual de Campinas, Campinas, SP, Brazil

**Keywords:** Contraceptives, oral, combined, Estradiol, Ethinylestradiol, Metrorrhagia, Spotting

## Abstract

**Objective:**

To review the real benefits of using combined oral contraceptives (COCs) containing natural hormones, also known as estradiol-containing COC (estradiol and estradiol valerate) in controlling abnormal uterine bleeding (AUB) compared to the already established data on treatment with combined pills containing ethinylestradiol (EE).

**Methods:**

Narrative review with analysis of studies published between 2010 and 2023, comparing the effects of EE and natural estrogen in the treatment of AUB, indexed in the PUBMED, WebOfScience, and Scielo databases.

**Results:**

A total of 342 articles were found in the selected databases. Of these, 235 articles were excluded because they were published before 2010 or lacked an explicit relationship with the topic; 107 articles were selected for title and abstract screening, of which 27 were fully read by the researchers, and 6 were included in the study. It was observed that the use of natural estrogens is effective in controlling abnormal uterine bleeding, often with fewer side effects and less cardiovascular and prothrombotic impact compared to ethinylestradiol (EE). However, some non-contraceptive benefits of EE, such as less water retention, improved skin oiliness, and acne, were not consistently observed with the use of natural estrogens.

**Conclusion:**

Estradiol-containing COC have proven to be effective in controlling AUB, in addition to reducing spotting and intermenstrual bleeding, with fewer undesirable side effects when compared to COCs with EE. On the other hand, COCs with estradiol and estradiol valerate less frequently present other non-contraceptive benefits.

## Introduction

Abnormal Uterine Bleeding (AUB) is characterized by blood loss originating from the uterine body with abnormalities in regularity, volume, frequency, or duration in non-pregnant women.^(1)^ It is a common complaint in gynecological consultations, affecting women's lives in various ways, particularly in psychological, sexual, emotional, and professional contexts, significantly impairing their quality of life.^(1,2)^ According to the International Federation of Gynecology and Obstetrics (FIGO), cycles lasting between 24 and 38 days, menstrual flow that persists for more than 8 days, and when considered by the woman (self-perception) as increased, are classified as abnormal (Chart 1).^(3)^ Also, absence of menstrual bleeding for more than 90 days is also considered an abnormalitie (amenorrhea). It is observed that the criteria for the diagnosis of AUB increasingly value the woman's perception of her own menstruation, with recent recommendations stating that treatments should aim not only to control the amount of blood lost but also to improve quality of life with the minimum possible side effects.^(4)^

**Chart 1:** Criteria for normal menstrual cycles according to the definitions of the International Federation of Gynecology and Obstetrics (FIGO-2018)

Frequency	Absent: amenorrhea Frequent: < 24 days Normal: 24-38 days Infrequent: > 38 days
Regularity	Regular variation: < 9 days Cycle variation: > 10 days
Duration	Prolonged: > 8 days Normal: < 8 days
Flow volume	Patient determined (self-perception)
Intermenstrual Bleeding	None Random Cyclic (predictable)
Unscheduled Bleeding on Progestin + Estrogen Gonadal Steroids (birth control pills, rings, patches or injections)	Not applicable None Present

Source: Adapted from Munro et al. (2018)^(3)^

Pharmacological or medical treatment is considered first-line therapy in most cases of AUB due to lower morbidity and less impact in reproductive future when compared to surgical treatment (especially in cases of hysterectomy and myomectomy, more complex surgeries), given that it is a condition affecting women of reproductive age.^(1)^ Combined oral contraceptives (COCs) containing estrogen and progestogen are widely used in various gynecological conditions due to their practicality and therapeutic success. In terms of controlling uterine bleeding, evidence suggests that COCs can reduce blood loss by 35 to 72%, making them an option for most structural and non-structural menstrual disorders, provided contraindications are respected. However, some formulations of these contraceptives may be associated with systemic side effects that can reduce adherence to treatment, particularly those containing ethinylestradiol (EE).^(1)^

EE is a synthetic hormone that undergoes slow metabolism, primarily by the liver. During its absorption and first-pass hepatic effect, an average bioavailability of approximately 45% is achieved, with wide interindividual variation (20 to 65%). Additionally, EE binds nonspecifically to serum albumin (approximately 98%), induces an increase in sex hormone-binding globulin (SHBG) concentrations, and promotes significant changes in the coagulation system. Data show that EE is capable of increasing the production of procoagulant factors (fibrinogen, VII, VIII, IX, X, XI, and XII) and reducing the action of natural anticoagulants (protein S, protein C, and antithrombin), facilitating the development of thromboembolic events in COC users containing this molecule.^(5-7)^ EE can also be associated with cardiovascular system dysfunction, with increased blood pressure in certain situations, as well as disruptions in lipid and glycemic profiles, with decreased insulin sensitivity described in women who use this medication long-term, particularly in those with associated comorbidities.^(8)^ Therefore, the potential use of other oral contraceptives with fewer systemic and metabolic effects is increasingly being discussed, with attention given to formulations containing hormones molecularly similar to endogenous hormones, known as natural hormones, for managing AUB.

Natural hormones, containing estradiol or estradiol valerate, are products approved for hormone therapy by regulatory agencies, such as the Food and Drug Administration (FDA) in the United States (USA) and the Brazilian Health Regulatory Agency (ANVISA) in Brazil. They have registered names that contain products chemically identical to the hormones produced by women (primarily ovarian hormones), such as estradiol.^(6)^ In recent years, new combinations containing estradiol have been developed, such as the association of estradiol valerate and dienogest (DNG) and estradiol (E2) with nomegestrol acetate (NOMAC). Data demonstrate greater tolerability of these formulations, with fewer side effects, and a significant reduction in menstrual bleeding during use, including an FDA-approved indication for this purpose in the USA in 2012.^(9)^ In Brazil, the indication for reducing menstrual flow is included in the package insert, but there are still few publications reviewing the effects of these medications specifically for AUB. The aim of this study is to review the real benefits of using COCs containing estradiol and estradiol valerate in controlling AUB compared to the established data on the use of EE for this purpose.

## Methods

A narrative review was conducted, analyzing studies published between 2010 and 2023 and indexed in the PUBMED, WebOfScience, and Scielo databases, with the aim of analyzing the effects of ethinylestradiol and natural estrogen in the treatment of abnormal uterine bleeding. This period was chosen based on criteria related to the relevance and currentness of scientific evidence, considering that after 2010 there were significant advances in the development and use of COC. The article search was conducted between October 2023 and February 2024, using the following descriptors: "estradiol valerate"; "abnormal uterine bleeding"; "heavy menstrual bleeding"; "estradiol"; "dienogest"; "NOMAC"; "ethinylestradiol"; "combined contraceptives". Articles that did not address the use of combined pills for AUB, did not cite ethinylestradiol or natural estrogen, or focused on the use of combined pills for other purposes and did not fall within the selected publication period (2010–2018) were excluded from the review. Selected articles addressed the concept of AUB and its causes, the pharmacological characteristics of ethinylestradiol and natural estrogen, and the practical applications of combined pills in abnormal uterine bleeding.

## Results

**Figure 1:**
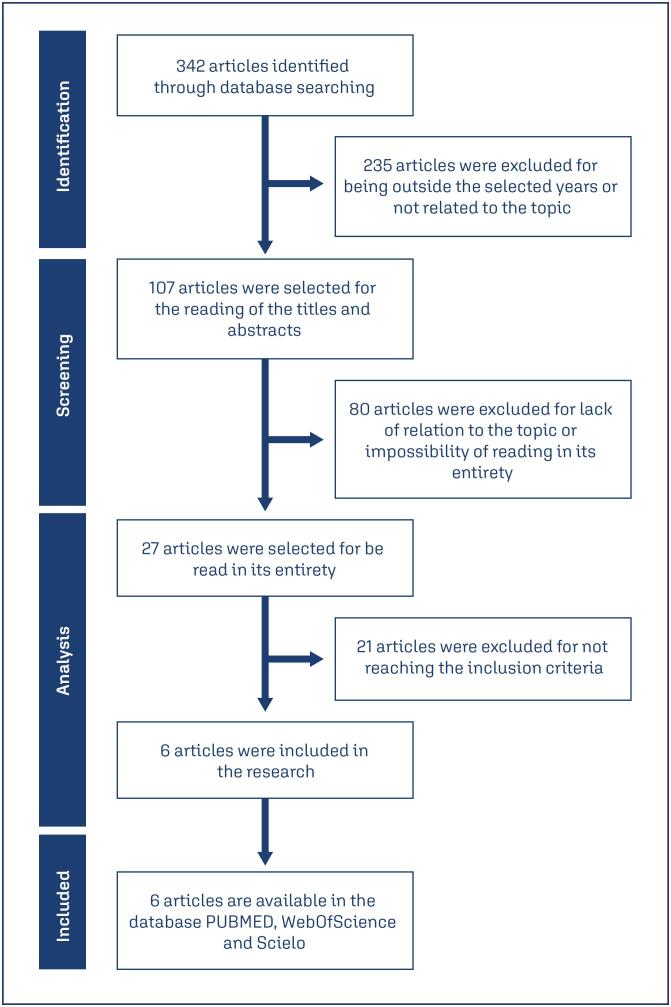
Flowchart of the review and inclusion of articles for the study

A total of 342 articles were found in the selected databases. Of these, 235 were excluded because they were published before 2010 or lacked an explicit relationship with the topic; 107 articles were selected for title and abstract screening, of which 27 were fully read by the researchers, and 6 were included in the study (Figure 1)

The literature review data demonstrated that the use of natural estrogens in COCs effectively controls vaginal bleeding, often with fewer side effects compared to ethinylestradiol (EE), as shown in chart 2. However, some non-contraceptive benefits related to the use of EE, such as improvements in skin oiliness, acne control, and reduced water retention, were less frequently associated with the use of natural estrogens. Additionally, the progestogens associated with estradiol or estradiol valerate (nomegestrol acetate and dienogest) were also associated with greater or lesser adherence to treatment (Chart 3).^(8-10)^

**Chart 2:** Summary reviewed studies findings

Study reference	Population	Intervention	Comparison	Outcome
Kangasniemi et al. (2020)^(9)^ J Clin Endocrinol Metab	56 women	Use of COCs containing either EV + DNG or EE + DNG	Use of DNG only	EV + DNG and DNG only had a neutral effect on inflammation and lipids, while EE + DNG increased both hs-CRP and PTX-3 levels as well as triglycerides and HDL
Wang et al. (2016)^(11)^ Int J Epidemiol	5841 women	Women using combined oral contraceptive pills (COCPs) or progestin-only contraceptives (POCs)	Who did not use hormonal contraception	Characteristics of POC users were broadly similar to those of non-users, whereas COCP users tended to be younger, leaner and consumed more alcohol than the other two groups
Haarala et al. (2009)^(12)^ Scand J Clin Lab Invest	1257 women	Women who use COCs	Those who do not use any hormonal contraceptives (non-users)	Our Study suggests that use of COCs alters the metabolic determinants and genetic regulation of CRP
Mansour et al. (2011)^(13)^ Eur J Contracept Reprod Health Care	On average, each of the 95 centres recruited between 20 and 60 women.	NOMAC/E2 users	DRSP/EE users	These data show that NOMAC/E2 provides high contraceptive efficacy with acceptable cycle control as well as an overall adverse event profile similar to that of DRSP/EE
Westhoff et al. (2012)^(14)^ Obstet Gynecol	2.281 women	Women who received nomegestrol acetate and 17ß-E2	Women who received drospirenone and ethinyl E2	Nomegestrol acetate and 17ß-E2 were well tolerated and provided excellent contraceptive efficacy and acceptable cycle control
Ågren et al. (2011)^(15)^ Eur J Contracept Reprod Health Care	121 women	Women who received NOMEC/E2	Women who received LNG/EE	The monophasic COC NOMAC/E2 had less influence on haemostasis, lipids and carbohydrate metabolism than the COC LNG/EE

**Chart 3:** Summary of COC's main characteristics

COC with Ethinylestradiol (EE)	COC with estradiol valerate/ estradiol
Increase production of procoagulant factors (fibrinogen, VII, VIII, IX, X, XI, and XII) mbin), Impact on lipid profile Non-contraceptive benefits: improvements in skin oiliness, acne control, and reduced water retention.	Lower risk of deep vein thrombosis (DVT), Less impact on lipid and hemostatic profiles Minimal SHBG impact Colateral effects: Spotting and acne

Dienogest (DNG) is a tissue-specific progestogen, with high affinity for endometrial tissue, that inhibits ovulation without suppressing gonadotropin production.^(2)^ Based on this, countries like Germany began producing pills that combined DNG with ethinylestradiol. More recently, however, EE was replaced by estradiol valerate to achieve better menstrual cycle control. This change resulted in a reduction in spotting and intermenstrual bleeding.^(10)^ However, some side effects were reported by patients using the formulation with estradiol valerate and DNG, such as weight gain, acne, and headaches, which may not occur in patients using other combined pills. On the other hand, the use of contraceptives that combine DNG and estradiol valerate was associated with a lower risk of developing deep vein thrombosis (DVT), as this combination has less impact on lipid and hemostatic profiles compared to those containing EE^(10)^ Therefore, for women with intermenstrual bleeding that negatively impacts their quality of life and who have higher metabolic and cardiovascular risks, this formulation may be a better option. In contrast, although there is a slight increase in SHBG with the use of estradiol and a mild antiandrogenic effect of dienogest, when compared to compositions with ethinylestradiol, the effect on signs of excess androgens such as hirsutism and androgenetic alopecia is smaller.^(16)^

Nomegestrol acetate (NOMAC) is a progestogen with a favorable tolerance profile and neutral effects on metabolism. It exerts an antiestrogenic effect at the endometrial level and has partially antiandrogenic activity.^(12)^ Its isolated use has already been employed for AUB treatment, with literature data demonstrating improvements in bleeding volume and intermenstrual bleeding in approximately 81% of cases.^(17,18)^

## Discussion

Some side effects were reported by patients using the formulation with estradiol valerate and DNG, such as weight gain, acne, and headaches, which may not occur in patients using other combined pills. On the other hand, the use of contraceptives that combine DNG and estradiol valerate was associated with a lower risk of developing deep vein thrombosis (DVT), as this combination has less impact on lipid and hemostatic profiles compared to those containing EE. Therefore, for women with intermenstrual bleeding that negatively impacts their quality of life and who have higher metabolic and cardiovascular risks, this formulation may be a better option. In contrast, although there is a slight increase in SHBG with the use of estradiol and a mild antiandrogenic effect of dienogest, when compared to compositions with ethinylestradiol, the effect on signs of excess androgens such as hirsutism and androgenetic alopecia is smaller.

COCs that combine estradiol with NOMAC have gained prominence in recent years, with studies showing that their use was not associated with changes in lipid, glycemic profiles, or blood pressure levels, nor was there a significant increase in prothrombotic factors compared to pills containing EE.^(18)^ In terms of AUB control, approximately 7 out of 10 women using this formulation experienced only spotting, with significant improvement in complaints of increased menstrual volume. However, side effects such as acne and headaches may occur.^(13-15)^

Despite the need for caution in prescribing any contraceptive containing estrogens, whether for family planning or AUB control, the development of new combinations is highly relevant. COCs with estradiol valerate and DNG, and estradiol and NOMAC, present a safer metabolic profile, with fewer impacts on lipid, glycemic, and thrombotic levels, in addition to significantly reducing complaints of abnormal uterine bleeding. Therefore, especially for women with AUB who present high cardiovascular and metabolic risks, the use of pills with natural estrogens may be an interesting option; however, more studies are needed to confirm this safety profile.^(11-13)^ Although this is a narrative review, a study design that has limitations, it is one of the few publications on the use of this type of medication specifically for the treatment of AUB, which should be considered.

## Conclusion

The introduction of natural hormones, such as estradiol valerate and estradiol, in the composition of combined oral contraceptives has shown positive results in controlling abnormal uterine bleeding compared to synthetic hormones (ethinylestradiol). Although COCs with EE are effective in controlling abnormal uterine bleeding, their use may result in side effects and increased cardiovascular risk. Estradiol-containing COC have also been shown to be effective in menstrual cycle control, in addition to reducing spotting and intermenstrual bleeding. On the other hand, COCs with estradiol and estradiol valerate less frequently offer other non-contraceptive benefits.
